# Comparison of outcomes between surgery and chemoradiotherapy after endoscopic resection for pT1a-MM with lymphovascular invasion or pT1b esophageal squamous cell carcinoma: Japanese multicenter propensity score-matched study

**DOI:** 10.1007/s00535-024-02188-7

**Published:** 2024-12-03

**Authors:** Yoshinobu Yamamoto, Ryu Ishihara, Hirofumi Kawakubo, Michiko Nishikawa, Sachiko Yamamoto, Tomohiro Kadota, Seiichiro Abe, Masao Yoshida, Tsutomu Tanaka, Hiroaki Nagano, Hiroyoshi Nakanishi, Tetsuya Yoshizaki, Kotaro Waki, Akiko Takahashi, Yoshiyasu Kitagawa, Kenichi Mizuno, Kenro Kawada, Yoshiyasu Kono, Chikatoshi Katada, Takashi Hashimoto, Yasuaki Nagami, Toshiyuki Yoshio, Toshio Shimokawa, Keiji Nihei, Kazuo Koyanagi, Ken Kato, Tomonori Yano, Manabu Muto, Yuko Kitagawa

**Affiliations:** 1 Department of Gastrointestinal Oncology, Hyogo Cancer Center, 13-70 Kitaoji-Cho, Akashi, Hyogo 673-8558 Japan; 2 Department of Gastrointestinal Oncology, Osaka International Cancer Institute, 3-1-69, Otemae, Chuo-Ku, Osaka, 541-8567 Japan; 3 Department of Surgery, Keio University School of Medicine, Tokyo, Japan; 4 Department of Gastroenterology and Endoscopy, National Cancer Center Hospital East, Kashiwa, Japan; 5 Endoscopy Division, National Cancer Center Hospital, Tokyo, Japan; 6 Division of Endoscopy, Shizuoka Cancer Center, Shizuoka, Japan; 7 Department of Endoscopy, Aichi Cancer Center Hospital, Nagoya, Japan; 8 Department of Gastroenterological, Brest and Endocrine Surgery, Yamaguchi University Graduate School of Medicine, Yamaguchi, Japan; 9 Department of Gastroenterology, Ishikawa Prefectural Central Hospital, Ishikawa, Japan; 10 Department of Gastroenterology, Kobe University School of Medicine, Hyogo, Japan; 11 Department of Gastroenterology and Hepatology, Faculty of Life Sciences, Kumamoto University, Kumamoto, Japan; 12 Department of Endoscopy, Saku Central Hospital Advanced Care Center, Nagano, Japan; 13 Endoscopy Division, Chiba Cancer Center, Chiba, Japan; 14 Division of Gastroenterology and Hepatology, Graduate School of Medicine and Dental Science, Niigata University, Niigata, Japan; 15 Department of Esophageal Surgery, Tokyo Medical and Dental University, Tokyo, Japan; 16 Department of Gastroenterology and Hepatology, Faculty of Medicine, Dentistry and Pharmaceutical Sciences, Okayama University, Okayama, Japan; 17 Department of Gastroenterology, Kitasato University School of Medicine, Sagamihara, Japan; 18 Department of Esophageal and Gastroenterological Surgery, Graduate School of Medicine, Juntendo University, Tokyo, Japan; 19 Department of Gastroenterology, Osaka Metropolitan University Graduate School of Medicine, Osaka, Japan; 20 Department of Gastroenterology, Cancer Institute Hospital, Japanese Foundation for Cancer Research, Tokyo, Japan; 21 Department of Medical Data Science, Graduate School of Medicine, Wakayama Medical University, Wakayama, Japan; 22 Department of Radiation Oncology, Osaka Medical and Pharmaceutical University, Osaka, Japan; 23 Department of Gastroenterological Surgery, Tokai University School of Medicine, Kanagawa, Japan; 24 Department of Head and Neck, Esophageal Medical Oncology, National Cancer Center Hospital, Tokyo, Japan; 25 Department of Therapeutic Oncology, Kyoto University Graduate School of Medicine, Kyoto, Japan

**Keywords:** Esophageal squamous cell carcinoma, Endoscopic resection, Additional treatment, Submucosal invasion, Lymphovascular invasion

## Abstract

**Background:**

Lymphovascular invasion (LVI) or pT1b is noncurative after endoscopic resection (ER) for esophageal squamous cell carcinoma (ESCC), and therefore surgery or chemoradiotherapy (CRT) is recommended. However, there has been debate regarding which treatment has better outcomes and whether individual risks should be considered.

**Methods:**

This was a multicenter, retrospective study conducted at 65 hospitals in Japan. The inclusion criteria were patients with ESCC who underwent ER between January 2006 and December 2015, with pT1a-muscularis mucosa (MM) with LVI or pT1b, with negative vertical margins, cN0M0, and who underwent surgery or CRT. A 1:1 propensity score-matched analysis was performed between two groups. The primary and secondary end points were overall survival (OS) and relapse-free survival (RFS). OS and RFS were also compared between two subgroups: low risk (pT1a-MM with LVI and pT1b without LVI) and high risk (pT1b with LVI) for metastatic recurrence.

**Results:**

Among 472 patients, 160 patients were selected from each group. The OS and RFS did not differ between surgery and CRT groups (hazard ratio, 0.887; *P* = .635 and hazard ratio, 1.036; *P* = .876, respectively). Subgroup analysis showed that CRT had a better prognosis in the low-risk group, and conversely, surgery had a better prognosis in the high-risk group. But these were not significant. The high-risk CRT group had a significant worse prognosis than the low-risk CRT group.

**Conclusions:**

In patients with noncurative ER for ESCC, surgery and CRT showed no difference in long-term outcomes. Indications for CRT in the high-risk group need further investigation because of poor prognosis.

**Supplementary Information:**

The online version contains supplementary material available at 10.1007/s00535-024-02188-7.

## Introduction

Endoscopic resection (ER) is one of the standard treatments for mucosal (T1a) esophageal squamous cell carcinoma (ESCC) [1–6]. Submucosal invasion (pT1b) or lymphovascular invasion (LVI) is noncurative ER, and therefore guidelines in many countries recommend surgery or chemoradiotherapy (CRT) as additional treatment because of the risk of metastasis [2–6]. However, there has been debate regarding which treatment has better clinical outcomes, and treatment options tailored to individual risk have not been clarified.

JCOG0508 evaluated the usefulness of prophylactic CRT in patients with pT1a-muscularis mucosa (MM) with LVI or pT1b ESCC with negative resected vertical margins based on the pathological diagnosis after ER [7, 8]. The results showed that the 5-year overall survival (OS) rate for prophylactic CRT was excellent, at 89.7% [95% confidence interval (CI): 81.1%−94.5%]. However, progression-free survival (PFS) in patients with pT1b combined with LVI was worse than that for patients without LVI.

Recent studies also reported that additional CRT for patients with pT1b combined with LVI after ER showed worse outcomes compared to additional surgery after ER [8–11]. However, these reports had small sample sizes, lacked details on irradiation fields and doses for CRT, lacked some patient background characteristics, lacked details on follow-up, and had biases in age, comorbidities, and double cancers. Therefore, whether surgery or CRT is more suitable as an additional treatment for patients with risk of metastasis has not been adequately addressed. In addition, a risk-based precision treatment strategy might contribute to improving prognosis. The present study aimed to compare the clinical outcomes between surgery and CRT as additional treatment for patients with pT1a-MM combined with LVI or pT1b ESCC after ER.

## Methods

### Study design

This multicenter, retrospective study was conducted at 65 hospitals in Japan, 64 of which belong to the Japanese Clinical Oncology Group (JCOG). One hospital participated in this study for statistical analysis only. The study protocol was approved by the ethics committee of Hyogo Cancer Center on September 15, 2021 (approval no. R-905). The study was conducted from September 15, 2021, to January 27, 2023 (data collection from October 1, 2021, to March 31, 2022; data analysis from April 1, 2022, to January 27, 2023). This study was conducted in accordance with the Declaration of Helsinki and Ethical Guidelines for Medical and Biological Research Involving Human Subjects [12]. Information about the study for patients was made available on the websites of the hospitals participating in the study. All patients were allowed to opt out of the study at any time.

### Participants

Patient information was collected from the electronic medical records and treatment databases of 64 hospitals. This study used a two-step process to enroll the patients. The initial enrollment criteria were as follows: (1) underwent ER with en bloc resection for esophageal cancer at our hospitals between January 2006 and December 2015; (2) the histological type was confirmed as squamous cell carcinoma using ER specimens; (3) age at ER was between 20 and 80 years; (4) lesions were located in the thoracic esophagus to the abdominal esophagus (Japanese Classification of Esophageal Cancer, eleventh edition) [13]; (5) diagnosed pT1a-MM with LVI or deeper than pT1b on pathological findings after ER in each participating institution with or without immunostaining; (6) no prior history of radiotherapy to the cervicothoracic region; and (7) no metastatic lesions diagnosed by computed tomography. The exclusion criteria were as follows: (1) multiple cancers within 5 years prior to ER (except for a history of cancer equivalent to a 5-year relative survival rate of at least 95%); and (2) severe organ dysfunction, poorly controlled hypertension, or diabetes mellitus at the time of ER.

Secondary enrollment criteria were applied with reference to JCOG0502 (comparison of upfront surgery versus definitive chemoradiotherapy in patients with stage I ESCC) [14] and JCOG0508, as follows: (1) underwent ER with negative resected vertical margin; (2) underwent surgery or CRT as additional treatment after ER; (3) in patients who underwent surgery, esophagectomy with lymph node dissection in two to three regions was performed; 4) in patients who underwent CRT, the chemotherapy regimen was two courses of cisplatin (70–75 mg/m^2^)/fluorouracil (2800–4000 mg/m^2^), and radiotherapy was conducted at a dose of 40–50.4 Gy with prophylactic irradiation of regional lymph nodes, and (5) blood laboratory examination results before additional treatment met all of the following: white blood cells ≥ 3000 × 10^3^/μl, hemoglobin ≥ 10.0 g/dl, platelets ≥ 10.0 × 10^4^/μl, total bilirubin < 1.5 mg/dl, aspartate aminotransferase < 100 IU/l, alanine aminotransferase < 100 IU/l, and creatinine < 1.5 mg/dl. Irradiation techniques were determined at each hospital. Patients whose chemotherapy or radiotherapy was reduced from planned doses were also eligible. The secondary exclusion criterion was insufficient follow-up (no computed tomography or endoscopic examination after additional treatment).

### Outcomes

The primary end point was OS. The secondary end points were relapse-free survival (RFS), cause-specific survival (CSS), metastasis-free survival (MFS), esophagectomy-free survival (EFS), and adverse events (AEs). Relapse was defined as the finding of regional lymph node and/or distant recurrence or new ESCC of pT1a-MM or deeper. Esophageal recurrence was defined as local recurrence or new ESCC diagnosed as pT1a-MM or deeper. Metastasis was defined as regional lymph node and/or distant recurrence. In this study, computed tomography or positron emission tomography/computed tomography was mandatory to confirm metastasis. AEs were divided into early and late phases. The early phase was defined as “before initial discharge” for surgery and “before 30 days from treatment completion” for CRT. The late phase was defined as “after initial discharge” for surgery and “after 30 days from treatment completion” for CRT [14]. In the present study, the analysis was performed in patients with negative resected vertical margins. OS and RFS according to risk were also evaluated for the surgery and CRT groups. Based on previous reports, pT1b with LVI was defined as the high-risk group [8, 9, 15–18]. The patients were divided into two subgroups according to pathological diagnosis after ER based on risk for metastatic recurrence: a low-risk group (pT1a-MM with LVI and pT1b without LVI) and a high-risk group (pT1b with LVI).

### Statistical analysis

Categorical variables are expressed as frequencies with percentages, and continuous variables are expressed as mean values and standard deviations. A propensity score-matched analysis was used to eliminate bias in treatment selection between the surgery and CRT groups. The factors used for matching were age, sex, body mass index, pathological tumor depth, lymphatic invasion, venous invasion, tumor location, tumor circumference, number of lesions (single/multiple), white blood cells, hemoglobin, platelets, creatinine, and C-reactive protein. For matching, a 1:1, k-nearest neighbor (KNN) algorithm with non-reciprocal extraction was used, with a caliper of 0.25. To check after matching, standardized difference scores and p values were calculated for each of the factors used to calculate the propensity score (2-sample test for continuous variables and Fisher’s exact method for categorical variables). The Kaplan–Meier method was used to estimate survival curves for OS, RFS, CSS, MFS, EFS, and annual survival rates, and 95% CIs were calculated using Greenwood’s formula. Brookmeyer and Crowley’s method was also used to calculate 95% CIs for median survival. The log-rank test was used to compare groups, and hazard ratios and 95% CIs were calculated. The hazard ratio was calculated as the ratio of CRT to surgery. Fisher’s exact test was used to compare two groups for additional surgery and additional CRT, and logistic regression analysis was used to determine hazard ratios and 95% CIs for each group. In the subgroup analysis, the outcomes of additional surgery and additional CRT were compared in the same way.

## Results

### Patients’ profile

**Fig. 1:**
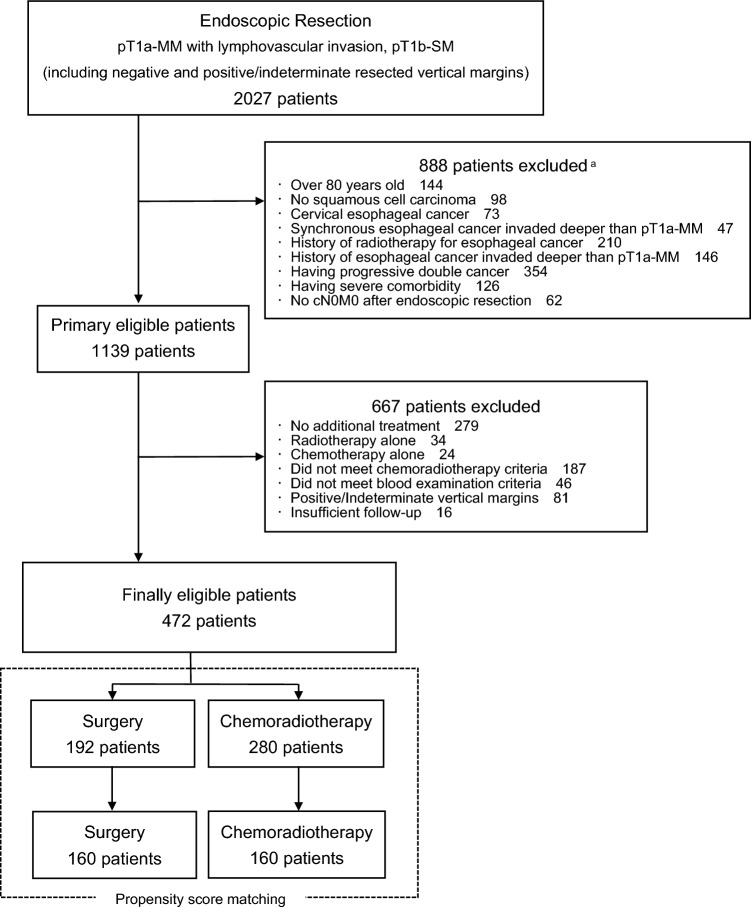
Study flowchart. **a** Some patients were excluded for one or more reasons

Between January 2006 and December 2015, 2027 patients underwent esophageal ER at 64 hospitals and were confirmed as having pT1a-MM with LVI or pT1b or deeper ESCC (Fig. 1). Of them, 1139 met the initial criteria, of which 472 met the secondary criteria and were analyzed in this study. Of the 472 patients, 192 underwent surgery, and 280 received CRT. The background characteristics of the 472 patients are shown in Table 1. Their median age was 65 years (range, 37–80 years); performance status was 0–1 in all patients; and the preoperative diagnosis was T1a-epithelial/lamina propria mucosa in 118 (25.0%) patients, T1a-MM/T1b-submucosa1 (SM1) in 282 (59.7%) patients, T1b-SM2 in 65 (13.7%) patients, and unknown in 7 (1.5%) patients. Endoscopic mucosal resection was performed in 48 (10.1%) patients, and endoscopic submucosal dissection was performed in 424 (89.9%) patients. The median follow-up period was 7.05 (range, 0.19–15.25) years. The follow-up rates at 5 years and 10 years were 94.1% (91.3% for surgery and 96.1% for CRT) and 36.8% (31.2% for surgery and 41.3% for CRT), respectively.

**Table 1:** Background characteristics of eligible patients

	Total *n* (%) (*n* = 472)
Median age, years; (range)	65 (37–80)
Sex	
Male	410 (86.9)
Female	62 (13.1
Performance status	
0–1	472 (100.0)
2–4	0 (0.0)
Smoking status	
Present	192 (40.7)
Former	202 (42.7)
Never	71 (15.0)
Unknown	7 (1.5)
Alcohol consumption	
Everyday	353 (74.7)
Sometimes	52 (11.0)
Never	55 (11.7)
Unknown	12 (2.5)
Preoperative tumor size, mm	
< 40	358 (75.8)
≥ 40	114 (24.1)
Preoperative tumor depth	
EP-LPM	118 (25.0)
MM-SM1	282 (59.7)
SM2	65 (13.7)
Unknown	7 (1.5)
Tumor location	
Upper thoracic	67 (14.2)
Middle thoracic	272 (57.6)
Lower thoracic	133 (28.2)
Tumor circumference	
< 1/2	320 (67.8)
≥ 1/2, < 1	140 (30.0)
Entire	12 (2.5)
Multiple lesions	
Absent	393 (83.3)
Present	79 (16.7)
Treatment	
Endoscopic mucosal resection	48 (10.1)
Endoscopic submucosal dissection	424 (89.9)
Pathological tumor size, mm	
< 40	370 (78.4)
≥ 40	102 (21.6)
Pathological tumor depth	
MM	112 (23.7)
SM1	122 (25.8)
SM2	238 (50.4)
Lymphatic invasion	
Negative	265 (56.1)
Positive	207 (43.9)
Vascular invasion	
Negative	333 (70.6)
Positive	139 (29.4)
Lateral resection margin	
Negative	377 (79.7)
Positive	45 (9.5)
Uncertain	50 (10.6)

*EP*, epithelial; *LPM*, lamina propria mucosa; *MM*, muscularis mucosa; *SM*, submucosa

### Propensity score matching

A 1:1 propensity score-matched analysis of 472 patients was performed, with 160 patients selected from the additional surgery group and 160 from the additional CRT group. Baseline characteristics of the surgery and CRT groups are shown in Table 2, with their ratios before and after matching. After propensity score matching, age, body mass index, lymphatic invasion, venous invasion, and tumor circumference were adjusted. The radiation dose in the 160 patients in the CRT group was 40–41.4 Gy in 138 patients (86.2%) and 42–50.4 Gy in 22 patients (13.8%). The chemotherapy dose per course was cisplatin 70 mg/m^2^, fluorouracil 2,800 mg/m^2^ in 153 patients (95.6%), and cisplatin 75 mg/m^2^, fluorouracil 4,000 mg/m^2^ in 7 patients (4.4%). Of the 160 patients in the surgery group, 56 underwent open surgery, and 104 underwent thoracoscopic surgery.

**Table 2:** Baseline characteristics of the surgery and CRT groups before and after propensity score matching

	Before matching			After matching		
Characteristics	Surgery group	CRT group	*p*-value	Surgery group	CRT group	*p* value
	*n* = 192	*n* = 280		*n* = 160	*n* = 160	
Age	63.55 [7.5319]	65.91 [7.2789]	0.001	64.28 [7.1207]	64.17[7.8816]	0.905
Sex			0.127			0.874
Male	161 (83.9)	249 (88.9)		136 (85.0)	138 (86.2)	
Female	31 (16.1)	31 (11.1)		24 (15.0)	22 (13.8)	
Body mass index	21.49 [2.831]	22.22 [3.0791]	0.009	21.57 [2.9241]	21.69 [3.0021]	0.707
Pathological tumor depth			0.031			0.587
MM	41 (21.4)	71 (25.4)		38 (23.8)	37 (23.1)	
SM1	62 (32.3)	60 (21.4)		40 (25.0)	48 (30.0)	
SM2	89 (46.4)	149 (53.2)		82 (51.2)	75 (46.9)	
Pathological tumor size, mm			0.500			0.895
< 40	146 (76.0)	221 (78.9)		124 (77.5)	122 (76.3)	
≥ 40	46 (24.0)	59 (21.1)		36 (22.5)	38 (23.7)	
Lymphatic invasion			0.006			1.000
Negative	93 (48.4)	172 (61.4)		83 (51.9)	83 (51.9)	
Positive	99 (51.6)	108 (38.6)		77 (48.1)	77 (48.1)	
Venous invasion			0.040			1.000
Negative	125 (65.1)	208 (74.3)		108 (67.5)	109 (68.1)	
Positive	67 (34.9)	72 (25.7)		52 (32.5)	51 (31.9)	
Tumor location			0.124			0.938
Upper thoracic	64 (33.3)	69 (24.6)		48 (30.0)	50 (31.2)	
Middle thoracic	103 (53.6)	169 (60.4)		89 (55.6)	89 (55.6)	
Lower thoracic	25 (13.0)	42 (15.0)		23 (14.4)	21 (13.1)	
Tumor circumference			0.009			0.866
< 1/2	115 (59.9)	205 (73.2)		102 (63.7)	106 (66.2)	
≥ 1/2, < 3/4	56 (29.2)	52 (18.6)		44 (27.5)	40 (25.0)	
≥ 3/4	21 (10.9)	23 (8.2)		14 (8.8)	14 (8.8)	
Number of tumors			0.531			1.000
Single tumor	157 (81.8)	236 (84.3)		132 (82.5)	133 (83.1)	
Multiple tumors	35 (18.2)	44 (15.7)		28 (17.5)	27 (16.9)	
White blood cells (/μl)	5672 [1500.7]	5814 [1532]	0.317	5673 [1534.7]	5779 [1610]	0.547
Hemoglobin (g/dl)	13.66 [1.3085]	13.98 [2.065]	0.062	13.7 [1.3351]	13.72 [1.4183]	0.897
Platelet (× 10^4^/μl)	22.02 [5.3677]	21.63 [5.6641]	0.452	21.88 [5.1394]	22.07 [5.7697]	0.752
Creatinine (mg/dl)	0.775 [0.1649]	0.789 [0.13733]	0.318	0.7746 [0.16084]	0.7793 [0.13219]	0.773
C-reactive protein (mg/dl)	0.1745 [0.3429]	0.2578 [0.67408]	0.115	0.1825 [0.36972]	0.2431 [0.64703]	0.305

Data are average [SD] or number (%)

### Survival analysis

**Fig. 2:**
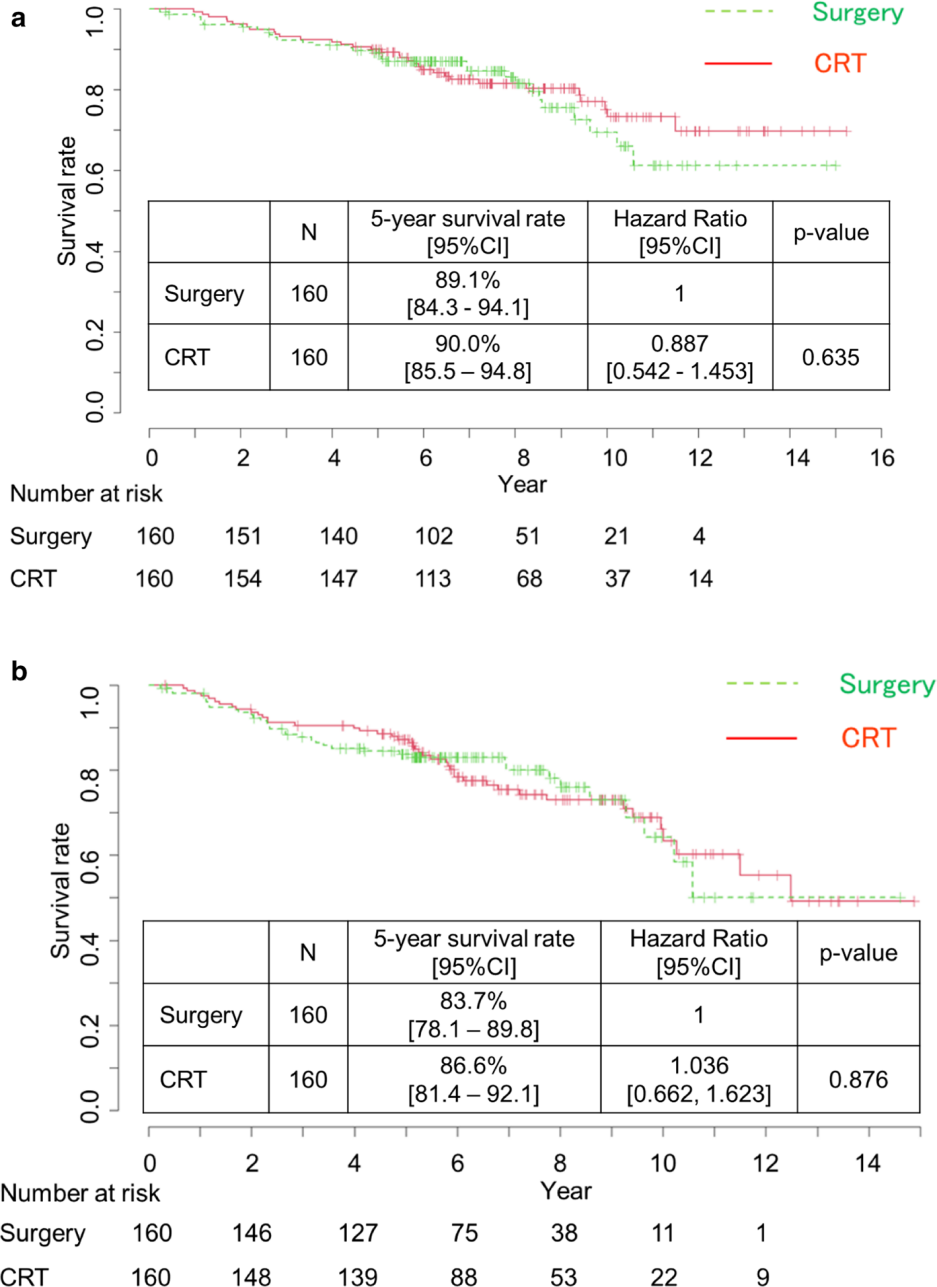
Overall survival and relapse-free survival in the surgery and CRT groups. **a** Overall survival. **b** Relapse-free survival

Figure 2 shows OS and RFS in the surgery and CRT groups. The OS did not differ between the surgery and CRT groups (hazard ratio, 0.887; 95% CI 0.542–1.453; *P* = 0.635). The 5-year OS was 89.1% (95% CI, 84.3–94.1%) and 90.0% (95% CI 85.5–94.8%) in the surgery and CRT groups, respectively. The RFS also did not differ between the surgery and CRT groups (hazard ratio, 1.036; 95% CI 0.662–1.623; *P* = 0.876). The 5-year RFS was 83.7% (95% CI, 78.1%−89.8%) and 86.6% (95% CI 81.4–91.2%) in the surgery and CRT groups, respectively. Supplementary Fig. 1 shows CSS and MFS in the surgery and CRT groups. Similarly, CSS and MFS did not differ between the surgery and CRT groups. The 5-year EFS in the CRT group was 90% (95% CI 0.855–0.948).

**Fig. 3:**
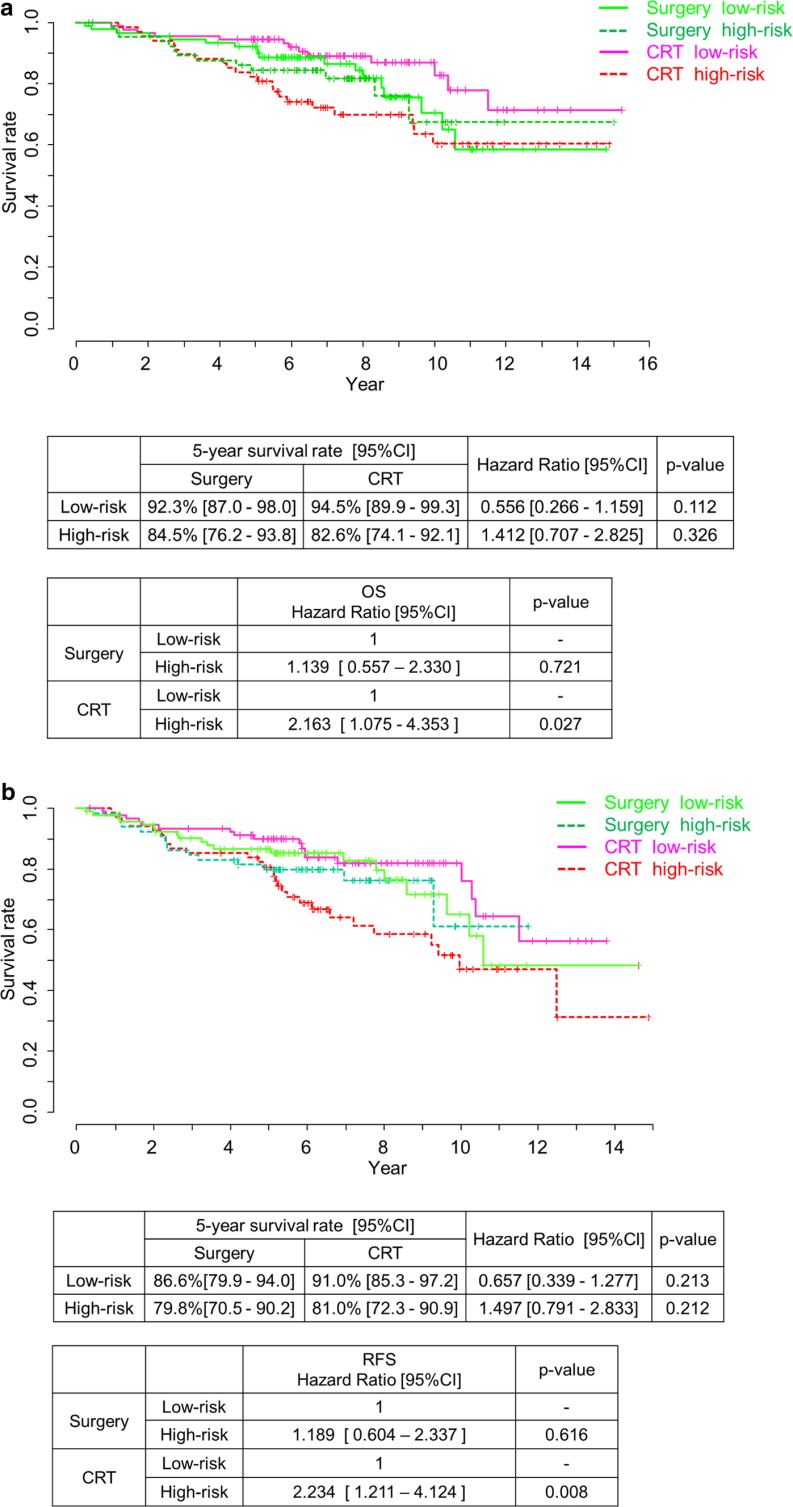
Overall survival and relapse-free survival in the surgery and CRT group divided into two subgroups (low-risk group and high-risk group). **a** Overall survival. **b** Relapse-free survival

Figure 3 shows OS and RFS for the low-risk and high-risk patients in the surgery and CRT groups. The hazard ratio was calculated as the ratio of CRT to surgery. In the low-risk group, the CRT group had better OS and RFS than the surgery group, but not significantly (hazard ratio, 0.556; 95% CI 0.266–1.159; *P* = 0.112, and hazard ratio, 0.657; 95% CI 0.339–1.277; *P* = 0.213, respectively). Conversely, in the high-risk group, the surgery group had better OS and RFS, but not significantly (hazard ratio, 1.412; 95% CI 0.707–2.825; *P* = 0.326, and hazard ratio, 1.497; 95% CI 0.791–2.833; *P* = 0.212, respectively). In the CRT group, OS and RFS were significantly worse in the high-risk group than in the low-risk group (hazard ratio, 2.208; 95% CI 1.096–4.444, *P* = 0.023 and hazard ratio, 2.141; 95% CI 1.172–3.906, *P* = 0.011).

Supplementary Table 1 shows OS and RFS divided into five categories according to depth and LVI: pT1a-MM with LVI, pT1b-SM1 without LVI, pT1b-SM1 with LVI, pT1b-SM2 without LVI, and pT1b-SM1 with LVI. There were no significant differences in OS and RFS between surgery and CRT in any category. In the low-risk group, hazard ratios of OS were 0.329, 0.541, and 1.102 in pT1a-MM with LVI, pT1b-SM1 without LVI, and pT1b-SM2 without LVI, respectively. OS of pT1a-MM with LVI was slightly better than pT1b without LVI. However, the results for these five subdivided categories are only informative data due to the small sample size.

Table 3 shows the recurrence patterns and periods in all patients, low-risk patients, and high-risk patients. In the surgery group, no esophageal recurrence was observed, and all 15 (9.4%) metastatic recurrences were observed within 5 years. The latest metastatic recurrence in the CRT group was observed 7.6 years after ER. In the CRT group, esophageal and metastatic recurrences were observed even after 5 years. In the CRT high-risk group, nine patients (13.4%) had esophageal or metastatic recurrence after 5 years.

**Table 3:** Recurrence patterns and periods in the surgery and CRT groups

All patients after PS matching	Surgery (*n* = 160)		CRT (*n* = 160)	
	Within 5 years	After 5 years	Within 5 years	After 5 years
Esophageal recurrence ^a^ n, (%)	0 (0.0)	0 (0.0)	2 (1.3)	6 (3.8)
Metastatic recurrence n, (%)	15 (9.4)	0 (0.0)	13 (8.1)	7 (4.4)
Low-risk patients after PS matching	Surgery (n = 93)		CRT (n = 93)	
	Within 5 years	After 5 years	Within 5 years	After 5 years
Esophageal recurrence ^a^ n, (%)	0 (0.0)	0 (0.0)	2 (2.2)	2 (2.2)
Metastatic recurrence n, (%)	6 (6.5)	0 (0.0)	6 (6.5)	2 (2.2)
High-risk patients after PS matching	Surgery (n = 67)		CRT (n = 67)	
	Within 5 years	After 5 years	Within 5 years	After 5 years
Esophageal recurrence ^a^ n, (%)	0 (0.0)	0 (0.0)	0 (0.0)	4 (6.0)
Metastatic recurrence n, (%)	9 (13.4)	0 (0.0)	7 (10.4)	5 (7.4)

*PS*, propensity score; *CRT*, chemoradiotherapy

^a^Esophageal recurrence was defined as local recurrence or new caner development diagnosed as pT1a-MM or deeper

In the CRT group, 138 patients received 40–41.4 Gy of radiotherapy (low-RT dose) and 24 patients received 45–50.4 Gy (high-RT dose). Death from any cause during the observation period was 27 (19.5%) in patients who received low-RT dose and 7 (31.8%) in patients who received high-RT dose. Death from esophageal cancer was 12 (8.7%) in patients who received low-RT dose and 4 (18.2%) in patients who received high-RT dose.

### Adverse events

AEs in the surgery and CRT groups are shown in Table 4 for the patients selected after matching. The major early AEs in the surgery group were anastomotic leakage, recurrent laryngeal nerve palsy, and lung infection. The major early AEs in the CRT group were anorexia and fatigue. In the surgery group, one patient died of a lung infection on postoperative day 87. The major delayed AEs in the surgery group were ileus and gastrointestinal stenosis. In the surgery group, one patient died of ileus 10.2 years after surgery. In the CRT group, there were few delayed AEs more than 30 days after treatment completion.

**Table 4:** Adverse events of the surgery and CRT groups

Adverse events in the surgery group *n* = 160 CTCAE version 5.0		Grade 3 (%)	Grade 4 (%)	Grade 5 (%)	Grade3-5 (%)
Early	Anastomotic leak	8.8	1.3		10.0
	Anastomotic stenosis		0.6		0.6
	Recurrent laryngeal nerve palsy	3.8	0.6		4.4
	Lung infection	3.1		0.6	3.8
	Postoperative hemorrhage	0.6			0.6
	Gastric obstruction	0.6			0.6
	Small intestinal stenosis	0.6			0.6
	Colonic perforation	0.6			0.6
	Enterocolitis infectious	0.6			0.6
	Skin infection	2.5			2.5
	Chylothorax	0.6			0.6
	Pleural effusion	0.6			0.6
Delayed	Ileus	2.5	1.3	0.6	4.4
	Gastrointestinal stenosis	11.3			11.3
	Recurrent laryngeal nerve palsy	0.6			0.6
	Lung infection	0.6			0.6

Adverse events in the CRT group *n* = 160 CTCAE version 5.0		Grade 3 (%)	Grade 4 (%)	Grade 5	Grade3-5 (%)
Early	Anorexia	3.8			3.8
	Nausea	0.6			0.6
	Vomiting	0.6			0.6
	Malaise	1.3			1.3
	Fever	0.6			0.6
	Esophagitis	0.6			0.6
	Esophageal stenosis	0.6			0.6
	Duodenal perforation		0.6		0.6
	Dermatitis radiation	0.6			0.6
Delayed	Pneumonitis	1.3			1.3
	Esophagitis	1.3			1.3

*CRT*, chemoradiotherapy

## Discussion

To compare the outcomes between additional surgery and additional CRT after ER for ESCC, this multicenter retrospective study eliminated bias in both groups as much as possible by a 1:1 propensity score-matched analysis. The results showed no differences between surgery and CRT in OS, RFS, CSS, and MFS in patients with pT1a-MM with LVI or pT1b after ER. Subgroup analysis showed that the CRT group had a favorable prognosis in the low-risk group, but the surgery group had a favorable prognosis in the high-risk group. Notably, the hazard ratio was reversed for the low-risk and high-risk groups in long-term outcomes between the surgery and CRT groups, although there was no significant difference.

In the multicenter, prospective, confirmatory study (JCOG0508), OS of the patients with pT1a-MM with LVI or pT1b after ER for ESCC was comparable to that of surgery if they received prophylactic CRT [7]. However, vascular invasion and insufficient chemotherapy (only one course) were the significant risk factors for worse PFS [8]. Recently, Katada et al*.* also reported that pSM1 and LVI were the risk factors for metastasis in patients with pT1a-MM or pT1b-SM1 after ER for ESCC. There were no differences in OS, RFS, and CSS between surgery and CRT, although at-risk cases (pSM1 and positive for LVI) need additional treatment after ER [15]. Hatta et al*.* reported that lymphatic invasion, pT1b-SM2, and positive vertical margin were the risk factors for metastatic recurrence in patients without additional treatment [16]. In contrast, since the prognosis was excellent (99.6%−100%) with salvage therapy even in the case of recurrence, they concluded that pT1a-pMM/T1b-SM1 cases with negative LVI and negative resected vertical margins could be followed with no treatment. From these reports, it has been stated that additional treatment after ER was needed for cases with LVI or SM1, but which treatment, surgery or CRT, is better remains controversial. From this study, it may be needed to develop future treatment strategies according to individual risks in patients with risks for metastasis after ER.

Low follow-up rates are an important issue when evaluating outcomes in clinical research. Especially for subjects with a good prognosis, a high follow-up rate is required. In the present study, the follow-up rate 5 years after ER was high (94.1%), and the median follow-up was sufficiently long (7.05 years). The long-term follow-up of the present study showed the differences in recurrence patterns and periods of the two groups. In the surgery group, no esophageal recurrence was observed, and all metastatic recurrences were observed within 5 years after ER. In the CRT group, however, esophageal and metastatic recurrences were observed more than 5 years after ER. In particular, nine (13.4%) patients in the CRT high-risk group had esophageal recurrence or metastatic recurrence more than 5 years after ER. These findings are important in considering risk-based surveillance methods for each treatment modality.

Regarding irradiation technique, anterior–posterior opposed fields were used during the first half of the target period, and multi-field technique such as four fields were adopted for most cases during the latter half. However, because the present study was described in the protocol only to include the irradiation field of regional lymph nodes, irradiation was performed in the technique of each hospital. In the CRT group, 138 patients received 40–41.4 Gy of radiotherapy (low-RT dose) and 24 patients received 45–50.4 Gy (high-RT dose). Both deaths from any cause and esophageal cancer were more common in patients who received high-RT dose than in patients who received low-RT dose. However, the sample size of patients who received high-RT dose was small, so the recommendation about RT dose cannot be determined from this study.

Regarding AEs in the present study, two treatment-related deaths were observed in the surgery group. In contrast, CRT had no treatment-related deaths and few AEs because the patients were treated according to the JCOG0508 regimen.

This study had several limitations. First, this was a retrospective study. To reduce the bias in the patients’ background characteristics, propensity score-matched analysis was performed. Second, this study was conducted in high-volume hospitals in Japan, specifically in the JCOG study group. However, treatment strategies after ER for ESCC require specialized knowledge, experience, and skill, so that the data from such hospitals are important to standardize the treatment for such patients. Third, the present study was a cohort study based on pathological diagnoses at each institution, with no central pathological diagnoses. In addition, this study did not evaluate the degree of differentiation of ESCC. Fourth, in this study, the preoperative depth of most lesions was mucosal or cT1b-SM1 cancer. If the majority of patients were diagnosed with cT1b-SM2, the number of patients diagnosed with pT1b-SM2 would increase. Deep submucosal invasion often accompanies lymphovascular invasion, which would increase the proportion of high-risk patients. In that situation, there may be a significant difference in long-term outcomes between the surgery and CRT group. Therefore, applying this study to whole cT1b ESCC may require caution.

In conclusion, this multicenter retrospective study showed that the long-term outcomes did not differ between additional surgery and additional CRT after ER for patients with pT1a-MM with LVI or pT1b ESCC. However, since CRT for high-risk groups was associated with a poor prognosis, further investigation is needed.

## Supplementary Information

Below is the link to the electronic supplementary material.

Supplementary file 1 Supplementary Fig. 1 Cause-specific survival and metastasis-free survival in the surgery and CRT groups. (a) Cause-specific Survival (b) Metastasis-free Survival (c) Esophagectomy-free Survival (DOCX 82 KB)

Supplementary file 2 Supplementary Table 1 Overall survival and relapse-free survival in the surgery and CRT group divided into 5 categories according to depth and LVI (a) Overall Survival (b) Relapse-free Survival (DOCX 121 KB)

## Abbreviations

ER: Endoscopic resection
ESCC: Esophageal squamous cell carcinoma
LVI: Lymphovascular invasion
CRT: Chemoradiotherapy
MM: Muscularis mucosa
OS: Overall survival
CI: Confidence interval
PFS: Progression-free survival
JCOG: Japanese Clinical Oncology Group
RFS: Relapse-free survival
CSS: Cause-specific survival
MFS: Metastasis-free survival
EFS: Esophagectomy-free survival
AEs: Adverse events
SM: Submucosa
EP: Epithelial
LPM: Lamina propria mucosa
PS: Propensity score
