# Supplementary figures and images for: Comparison of outcomes between surgery and chemoradiotherapy after endoscopic resection for pT1a-MM with lymphovascular invasion or pT1b esophageal squamous cell carcinoma: Japanese multicenter propensity score-matched study
Source: J Gastroenterol. 2024 Dec 3;60(1):43–54. doi: 10.1007/s00535-024-02188-7 (PMC11717814; doi:10.1007/s00535-024-02188-7)

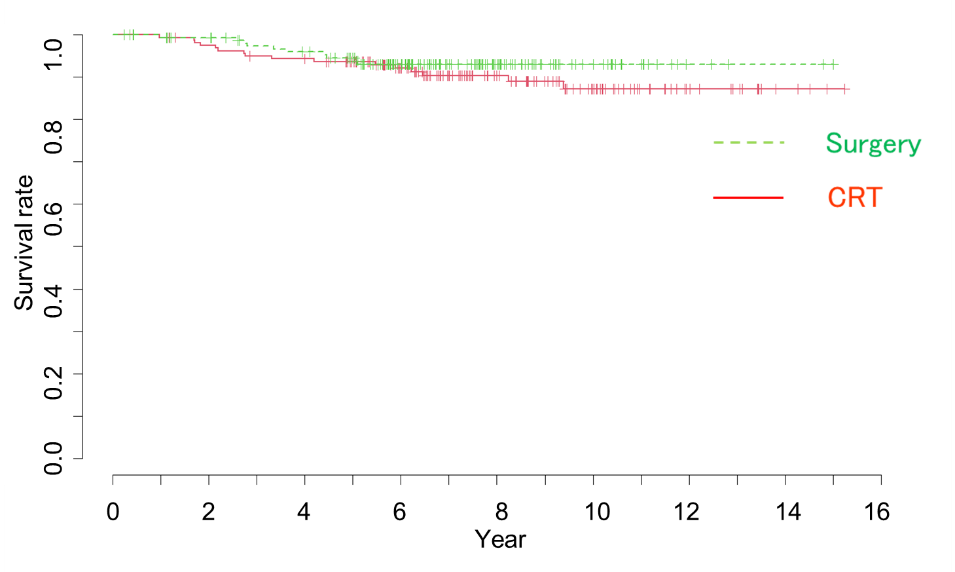


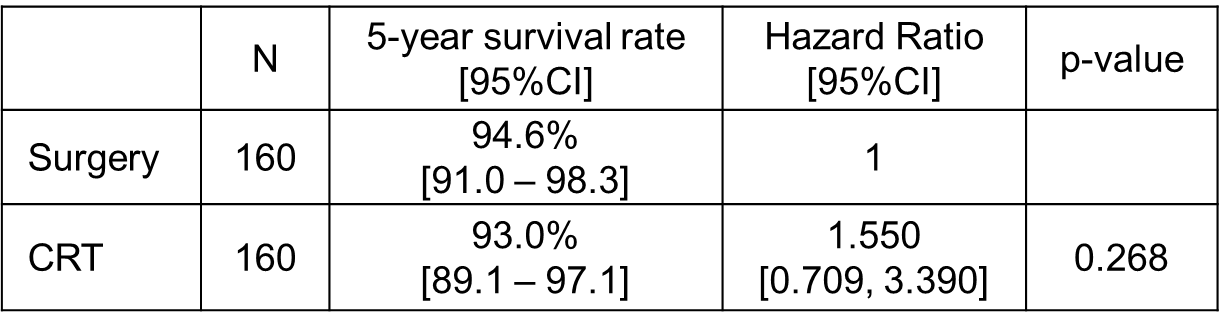


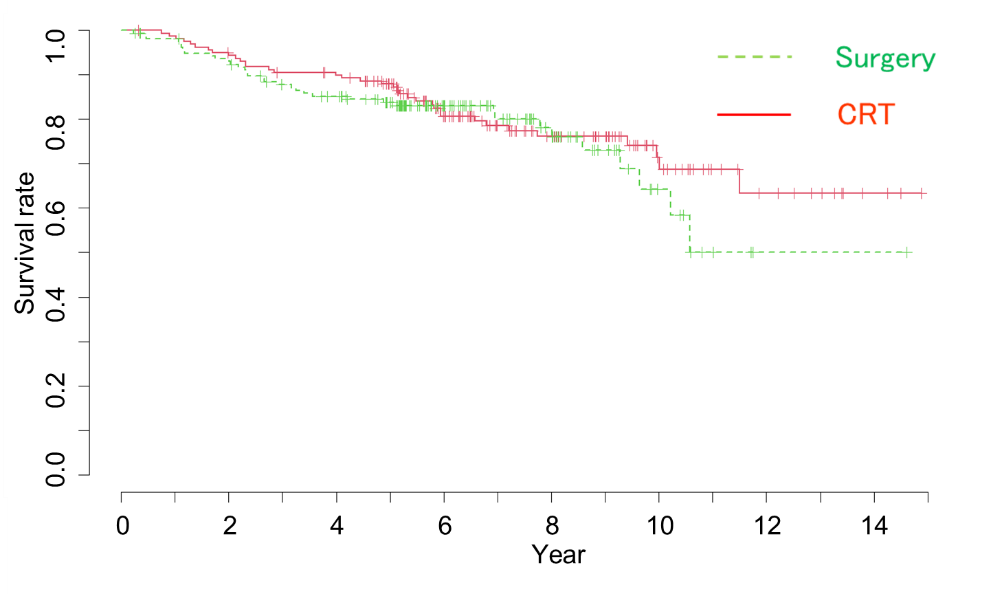


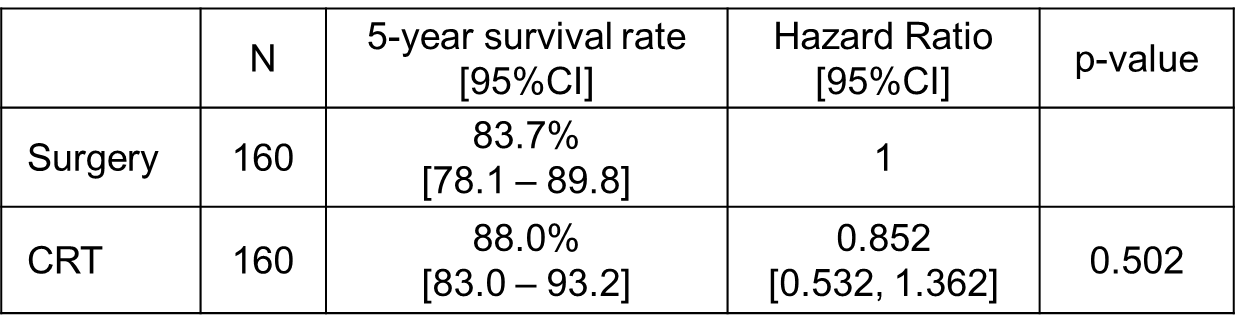


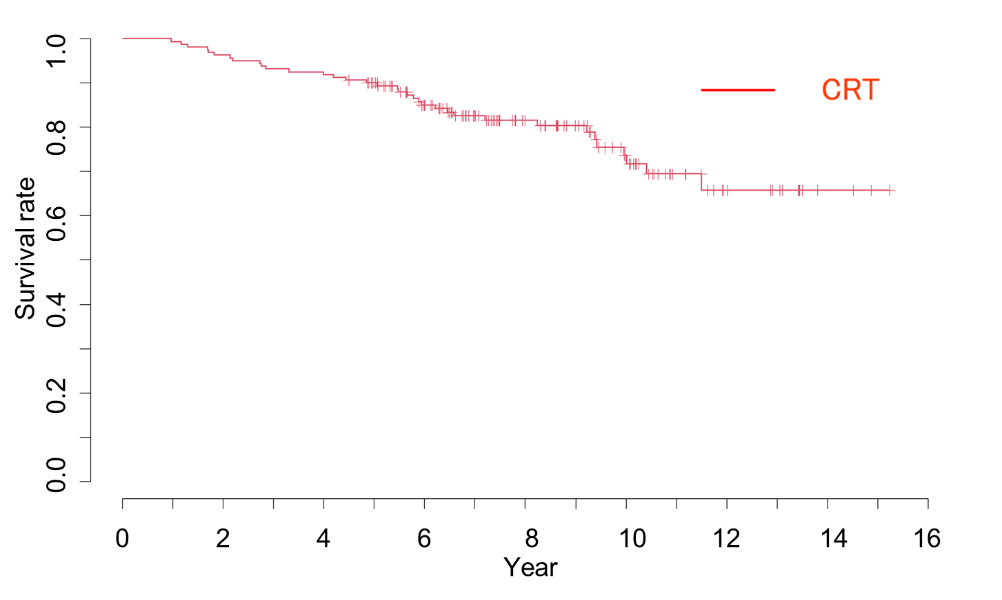


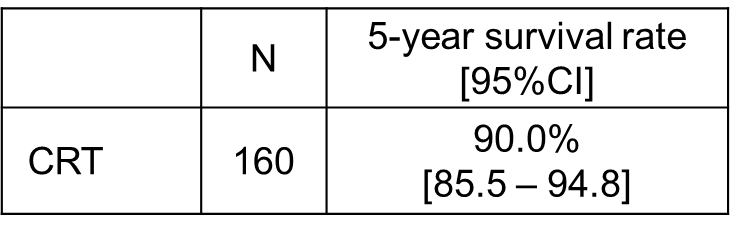

Supplement: Supplementary file 1 — Supplementary Fig. 1 Cause-specific survival and metastasis-free survival in the surgery and CRT groups. (a) Cause-specific Survival (b) Metastasis-free Survival (c) Esophagectomy-free Survival (DOCX 82 KB) [file 535_2024_2188_MOESM1_ESM.docx]

(a) Overall Survival


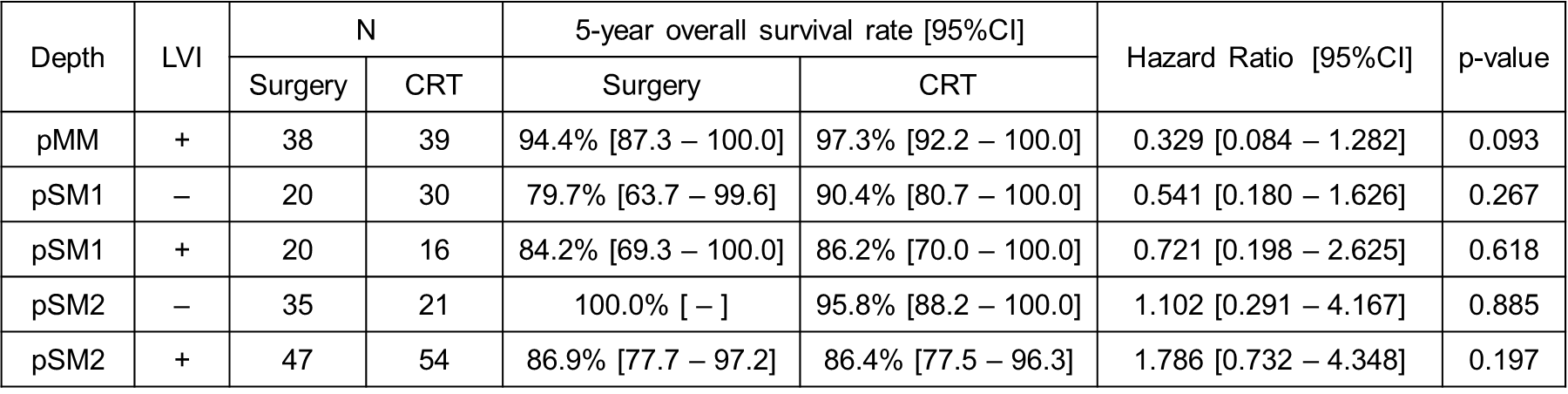


(b) Relapse-free Survival


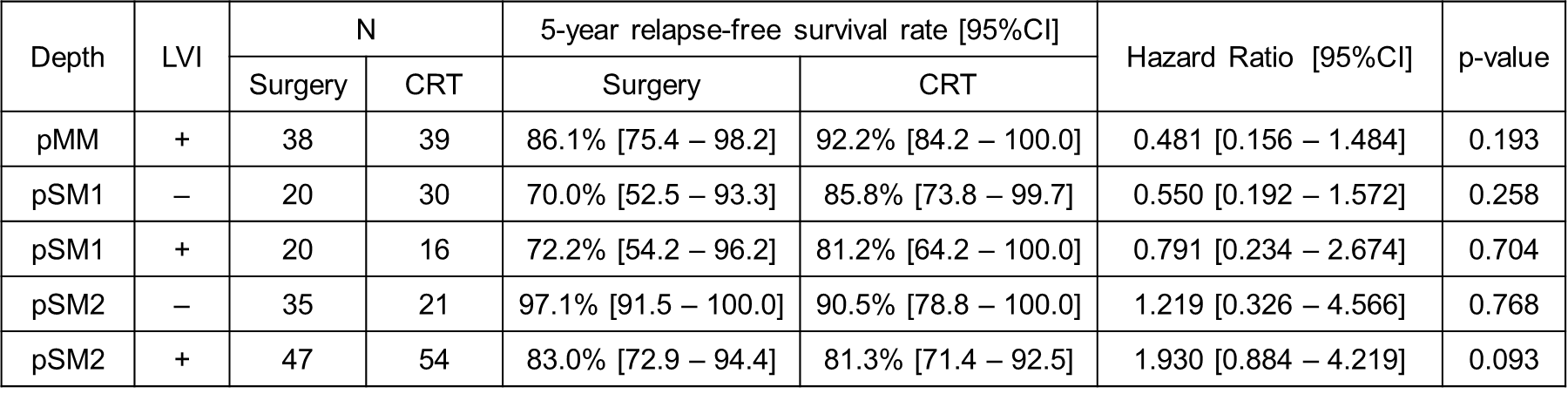

Supplement: Supplementary file 2 — Supplementary Table 1 Overall survival and relapse-free survival in the surgery and CRT group divided into 5 categories according to depth and LVI (a) Overall Survival (b) Relapse-free Survival (DOCX 121 KB) [file 535_2024_2188_MOESM2_ESM.docx]
